# Some Observations on the Use of Obstetrical Forceps

**Published:** 1912-06

**Authors:** H. R. Donaldson


### "SOME OBSERVATION ON THE USE OF OBSTETRICAL FORCEPS."

By H. R. Donaldson, A. B., M. D.

I do not expect in this paper to enter into a minute discussion of all phases of obstetrical work in which forceps are required. I simply wish to present some points which have impressed themselves upon my mind in actual experience, and hope that it may elicit some discussion which will be beneficial to all of us.

It might be interesting to say a few words with regard to the history of obstetrical forceps. The earliest account we have of the use of forceps was by Albucasis in the Eleventh Century. This forcep is cerated on the inner concave surface, and evidently was intended to be used on the dead foetus. The discovery of the obstetrical forcep is due to Dr. Peter Chamberlin, as most of you will recall, who lived in the latter part of the Sixteenth and early part of the Seventeenth Century. They were used by himself and his posterity for three or four generations before the family secret was finally given to the world. The Chamberlin forcep was a crude affair, short, having a concave cephalic curve. There have been any number of improvements made, but they can be classified under three heads: The first was making the forceps longer by increasing the length of the shaft. The second by giving the pelvic curve to the instrument, which greatly facilitated its use. The third, and in some respects the greatest improvement, was the addition of the axis traction by Taunier in 1877. The choice of forceps to be used depends very greatly on individual taste, and especially on being accustomed to one variety or another. Personally, I prefer the Taunier forcep. Recently, the Dewee forcep has come into rather general use and seems to be well liked. I have used this forcep, but prefer the Taunier. So much for the instrument itself.

With regard to the indications for the use of forceps, I would not presume sufficiently to go into those indications, as I know that all of you are familiar with them. I would like to add this observation, however, that it appears to me that more primiparae require the use of forceps than formerly. This possibly is due to our advanced civilization making a greater disproportion between the diameters of the head and pelvis. As a general rule, the forceps are indicated after two hours of second stage pains with no advance. When, however, the head is on the floor of the pelvis, we would hardly be justified in waiting more than half that time. Just when the border line is reached between interference and non-interference, is not easy to make clear by any hard and fast rule.

With regard to preparation of the patient, I wish especially to emphasize this point, and that is, that when it has been decided that forceps are necessary, it usually creates more or less a state of chaos in the sick room and among the family and attendants, and not infrequently the physician is not sufficiently painstaking in getting his patient in the proper position, whether across the bed or on a table, which is always better, if he has a mid-forceps delivery. The usual preparation with regard to aseptic should not be overlooked in any detail, and it is always best, if possible, to have a physician give the anaesthetic. I have found that when I had decided to complete the delivery by forceps, that by calling a physician, it usually gave me time to go about the preparation of the patient, sterilizing the instruments and other things that are necessary, and it most certainly relieves one's mind of the worry connected with an anaesthetic given by a novice.

Now, we come to one of the most important points which I wish to make, and that is, with regard to a correct diagnosis of the presentation. With the patient anaesthetized and in position and with a thoroughly dilated cervix which should always obtain, by introducing the entire hand, we accomplish not only the relaxation of the vulva, but are able to locate the posterior ear, which is absolutely essential, the Fontanelle, of course, having been located. With regard to the application of the forceps themselves, there are recognized two methods: the first, the pelvic, which has reference simply to introducing the left blade to the left side, and the right blade to the right side, without any regard to the fitting of the blades to the child's head. The better and equally as easy application is what is called the cephalic, with the hand in the vagina the posterior ear is located, the blade is introduced, using that hand as a guide. The other blade is introduced by changing hands and using the opposite hand as a guide, and by working the top blade into position it is usually very easily locked, so that the biparietal diameter corresponds to the center of the opening in the blades. We have then a method not only of traction without the danger of the blades slipping, but the handles themselves; especially, in occipito-posterior positions, makes us at all times a master of the situation with regard to the position of the head. Especially is this true when we have to depend upon rotation by the forceps. I might add just here that when this rotation is required, it is my practice to remove the forceps and re-apply them as they become inverted when the occiput is rotated anteriorally. When the head has been brought down in mid-forceps operation to the floor of the pelvis, and is bulging at the vulva, it has been my practice for some time to remove my Taunier forceps and apply short, low forceps especially adapted for that work. It is my opinion that you can control the head, reduce the per cent. and degree of perineal tears thereby, and in addition you give the parts an opportunity to relax while making the change. And just here, I would like to make this suggestion, that most operators are seized with a desire to complete the delivery just at this stage, and usually do so, very much more rapidly than should be done. I have spoken practically only with reference to mid and low forcep operations, as I do not believe high forceps should be undertaken unless a man has had considerable experience and everything is auspicious. With regard to applying forceps to the floating head, I have never had occasion to do this, and I would much prefer attempting delivery in some other way when the head refuses to engage.

In conclusion, I wish to rehearse the following points that should be particularly stressed:

First, that we should be conservative in our decisions to use the forceps, but should not hesitate unduly when the occasion arises.

Second, to go about the preparation for this operation with as much precision as we should about any other major operation.

Third, that whenever possible, the anaesthetic should be administered by a physician.

Fourth, that a correct diagnosis of the presentation be made, especially commending the use of the whole hand introduced into the vagina in making that diagnosis.

Fifth, the cephalic application of the forceps, the key to which is locating the posterior ear.

Sixth, that gentle and intermittent traction be made, and above all things, that sufficient time be taken in completing the delivery.

Seventh, I especially commend the use of the short forceps for the completion of the delivery.

